# Lactation at hot temperature: a test of heat dissipation limitation in mice divergently selected for basal metabolic rate

**DOI:** 10.1098/rsbl.2025.0048

**Published:** 2025-06-04

**Authors:** Sylwia Buczyńska, Aneta Książek, Sebastian Maciak, Paweł Brzęk, Catherine Hambly, John R. Speakman, Marek Konarzewski

**Affiliations:** ^1^ Faculty of Biology, University of Białystok, Białystok, Poland; ^2^ Institute of Biological and Environmental Sciences, University of Aberdeen, Aberdeen, United Kingdom, Aberdeen, UK; ^3^ Shenzhen Key Laboratory of Metabolic Health, Center for Energy Metabolism and Reproduction, Shenzhen Institutes of Advanced Technology, Chinese Academy of Sciences, Shenzhen, China

**Keywords:** artificial selection, basal metabolic rate, body temperature, lactation, heat dissipation limits, parental output

## Abstract

The HDL (heat dissipation limitation) hypothesis posits that mammalian energy budgets (SuSMR, sustained metabolic rate) are limited by the ability to dissipate metabolic heat. The HDL hypothesis has been tested in lactating mice but rarely systematically differs in SuSMR. Here, we used lines of laboratory mice divergently selected for basal metabolic rate (BMR) and effectively co-selected for SuSMR. We exposed lactating females to 23 and 30°C and manipulated their heat dissipation abilities by fur shaving. Exposure to 30°C did not affect the high BMR mice’s litter mass but increased litter mass in the low BMR mice. Fur shaving did not affect litter mass. However, it decreased body temperature (*T*_b_) by 0.2°C in the shaved mice, independent of line affiliation and ambient temperature. In both lines exposed to 30°C, the *T*_b_ increased by 0.2°C, while daily energy expenditure (a proxy of SuSMR) decreased by 20% and still was higher in the high BMR mice. These results do not support the HDL hypothesis. Low SuSMR individuals may benefit from higher ambient temperatures because of reduced costs of thermoregulation. It may change the course of natural selection towards reducing SuSMR and BMR.

## 1. Introduction

The heat dissipation limitation (HDL) hypothesis posits that long-term, sustained energy budgets (SuSMR, *sustained metabolic rate*) are limited by the ability to dissipate metabolic heat [1–3]. Numerous studies aimed at testing the HDL hypothesis were mainly focused on lactation, the most energetically demanding period in the life of female mammals, associated with increased food intake, increased metabolic rate and elevated body temperature [4–7].

Predictions of the HDL hypothesis have been tested using exposure of female mice with their pups to a low [8–11] or a high ambient temperature [2,12–15] and/or removing the insulation by shaving off the dams’ fur [6,16–19]. Both approaches focused on manipulating the rate of dissipation of metabolic heat generated during lactation. These studies have provided mixed support for the HDL theory, making the discussion about factors limiting the maximum rate of energy turnover still open.

As lactation is associated with an intensive energetic effort over a relatively long time scale (SuSMR), females’ investment in their parental output, assuming unlimited energy availability, should be affected by heat dissipation constraints. To date, the HDL hypothesis has customarily been tested on animals that do not differ with respect to SuSMR throughout lactation. However, without innate differences in SusMR, the effects of experimental manipulation of ambient temperature or insulation layer may remain inconclusive. Here, we present an experimental test of the HDL hypothesis in two lines of laboratory mice divergently selected for low and high basal metabolic rate (L-BMR and H-BMR lines, respectively) and systematically differing with respect to SuSMR [20–22]. Apart from the over 50% higher BMR, the H-BMR mice consume 17% more food, have a higher mass of the small intestine, liver, kidneys and heart (by approximately 13%, 17%, 18% and 14%, respectively) [23–25], and by 0.6°C higher core body temperature at both 23 and 30°C than mice with the low BMR [26,27]. Moreover, at 23°C, the parental output of the H-BMR mice is also higher: they produce 64% more milk per 24 h with 26% higher lactose content, and their litters grow faster than pups from the L-BMR line [20,21]. Higher food intake and daily energy expenditure (DEE) result in higher SuSMR in the H-BMR mice, with no manifestation of the HDL, such as a reduction of the growth rate of pups [28]. Both lines do not differ with respect to body mass [23–25]. In contrast to studies based on other lines of selected laboratory mice (e.g. [29]), the between-line difference in BMR is accompanied by the same heat dissipation capacity, underlined by the lack of the between-line difference in the insulative properties of the fur [25,26,30].

We reduced the ability to dissipate heat by exposing lactating females of both lines to 30°C (and 23°C as a control). In choosing 30°C, we were guided by earlier studies (reviewed by Król *et al*. [31]) demonstrating that the exposure of lactating female rodents to 30°C causes a reduction of food consumption, milk production and the mass of young. Yet, exposure to 30°C, unlike higher temperatures, does not compromise animals’ welfare, resulting in, e.g. mothers’ mortality or infanticide (e.g. [3]). Finally, the temperature of 30°C falls within the upper range of environmental temperatures and thermal tolerance of wild mammals (fig. 1B in [7]) and, therefore, is ecologically and evolutionarily relevant.

We hypothesized that if the HDL holds, at 30°C, the reduced thermal gradient will limit heat dissipation, forcing animals to reduce their DEE and litter mass. Furthermore, we combined lactation at hot temperature with enhancement of heat dissipation capacity by shaving off the dorsal pellet of dams [16–18]. In agreement with the HDL, we predicted that fur shaving would relieve dams from HDL and increase DEE and litter mass in a line-dependent manner. Most importantly, we predicted that the adverse effect of high ambient temperature would be particularly prominent in the H-BMR mice because of their high SuSMR. Unlike most earlier studies [2,7,32,33], we monitored the effect of experimental manipulations on core body temperature (*T*_b_)—the key indicator of heat stress. We expected *T*_b_ to be the highest in the unshaved H-BMR mice at 30°C.

## 2. Material and methods

We used female laboratory mice (*Mus musculus*) from the 53rd generation of a long-term selection experiment designed to generate two lines with divergent basal metabolic rates (respectively, the L-BMR and H-BMR lines), as described previously [23–25]. Briefly, depending on the breeding success, we maintain 30−35 families in each selected line in subsequent generations. Whenever possible, no less than three randomly chosen males and three females from each family are subjected to measurements of BMR. Animals characterized by the highest (in the H-BMR line) or lowest (in the L-BMR line) body mass-corrected BMR are chosen as progenitors and continuously mated outside their families. Our selection lines are not replicated, but we repeatedly demonstrated that the between-line differences in the key selected trait, BMR, as well as correlated traits (chiefly food consumption, internal organs mass and growth rate of the pups), are more significant than those expected due to genetic drift alone and, therefore, represent a genuine effect of selection [20–24,34,35].

Before the experiment, randomly chosen females from both lines were implanted intraperitoneally with loggers (type DST nano-T, size 6 mm × 17 mm, weight 1.3 g, Star Oddi Logging Life Science, Iceland) to measure core body temperature (*T*_b_) every 10 min. After recovery from surgery, they were mated with non-sibling males from the same line as females. On the day of parturition, females from both lines were randomly exposed to 23 or 30°C—the temperature just below the upper critical temperature of the H-BMR mice [26,30].

From the second day of lactation, every 2 days, litter mass was monitored (a proxy of parental effort). On the sixth day of lactation, half of the mothers from both lines, at both ambient temperatures, were dorsally shaved to the skin with an electric clipper (Remington). The procedure was repeated on the 10th day of lactation to minimize the fur regrowth [16]. Mothers were not anaesthetized before the procedure, and the same handler shaved each female in 5 min.

Between the 12th and 14th day (a peak of lactation in our animal model) [20], we used the doubly labelled water method [36] to measure DEE, a proxy of SuSMR in our study. Females were injected intraperitoneally with water containing ^18^O and ^2^H isotopes, and the substrates were incorporated into metabolic processes. The initial (after the isotope equilibrium reached in the body) and final (48 h after dosing) blood samples were collected to calculate the elimination rates of isotopes in lactating females. Individual differences in isotope elimination rates within the 2 days of peak lactation were converted to values of DEE according to equations in [37] (for more method details, see the electronic supplementary material). The experiment was terminated on the 14th day of lactation, as later pups started to nibble solid food and did not exclusively rely on maternal milk [20].

Each female raised only a single litter during the experiment. We used a total of 57 experimental females with 12 L-BMR at 23°C (six with fur, seven shaved) and 18 L-BMR at 30°C (eight with fur, 10 shaved), as well 14 H-BMR at 23°C (seven with fur, seven shaved) and 13 H-BMR at 30°C (six with fur, seven shaved). Of the 57 experimental females (within each experimental group, females originating from a separate family), *T*_b_ datasets were collected for 25 mother mice with implanted loggers (13 and 12 from the L-BMR and H-BMR line, respectively). BMR of females from the H-BMR and the L-BMR line assessed before the implementation of loggers averaged 62.11 ± 1.10 and 39.83 ± 0.69 ml O_2_ h^−1^, respectively.

Differences in litter mass (on the 12th day of lactation), maternal *T*_b_ and DEE (between the 12th and 14th day of lactation) were tested by three-way ANOVA/ANCOVA, in which line affiliation (L-BMR or H-BMR), ambient temperature (23 or 30°C) and manipulation (shaved or unshaved) were the main fixed effects. Litter mass on the 12th day of lactation was applied as a covariate in the case of *T*_b_ and DEE analysis. When significant interactions involved the effect of ambient temperature on litter mass, maternal *T*_b_ and DEE in the main model, then we performed the analysis separately within 23 and 30°C using two-way ANOVA/ANCOVA with line affiliation and manipulation as the main effects and the same covariate, respectively, for a specific analysis. We also analysed a partial correlation between litter mass and *T*_b_ of mothers, controlling for the effect of ambient temperature. Non-significant interactions at *p* > 0.05 were dropped from the final model.

Differences in litter mass and maternal *T*_b_ and DEE are expressed as absolute values from ANOVA ± s.e. or as least square mean values from ANCOVA ± s.e., respectively. Significance was tested at *p* = 0.05. Statistical analyses were carried out using the STATISTICA v. 13.3 package.

## 3. Results and discussion

### (a) Litter mass

**Figure 1.**
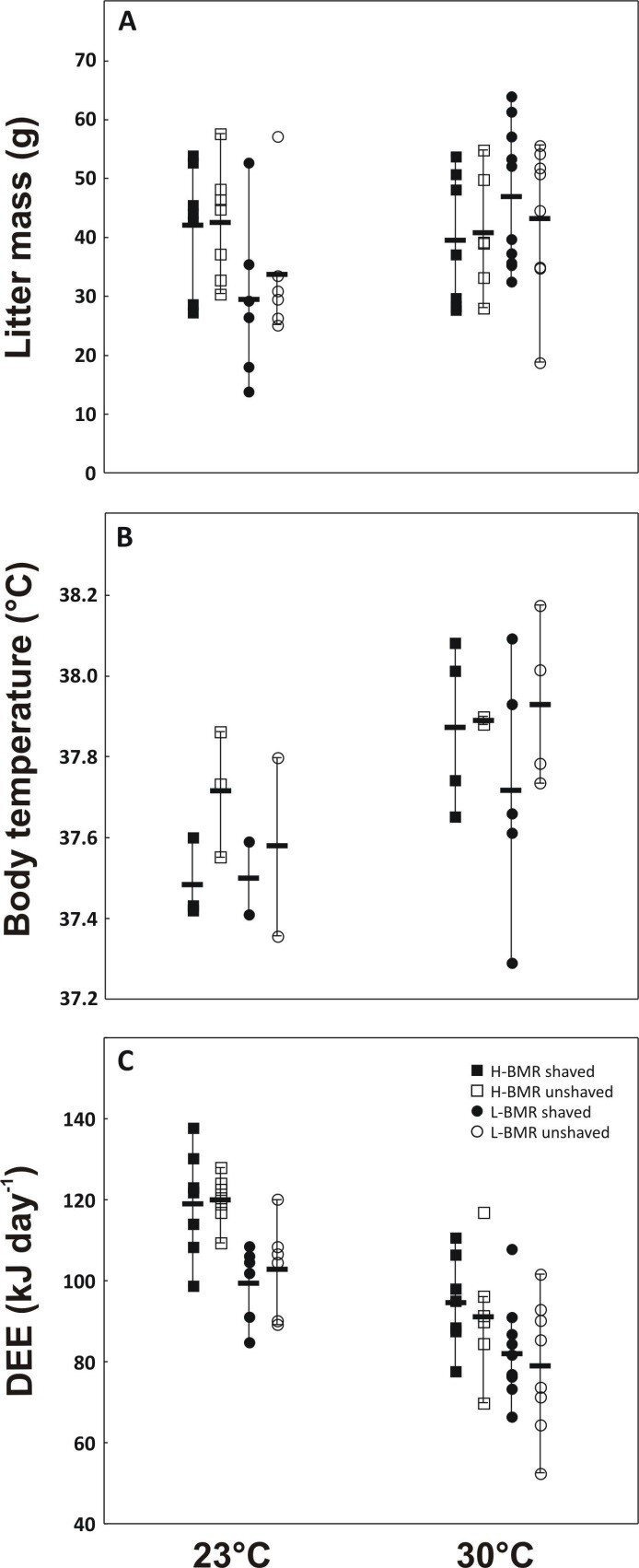
Litter mass on the 12th day of lactation (A), the mean core maternal *T*_b_ (B) and DEE over days 12−14 of lactation (C) in the L-BMR and H-BMR mice raising litters at temperatures of 23 and 30°C. Symbols represent individual animals, except for horizontal stretch denoting the mean.

According to the HDL hypothesis, we expected that the exposure of mothers and their pups to 30°C should decrease litter mass, particularly in the H-BMR line, which has higher SuSMR. Thus, we expected the response to ambient temperature to be line-dependent. Indeed, the line × ambient temperature interaction was significant (table 1). Comparisons carried out separately within 23 and 30°C revealed that litters of the H-BMR mice were heavier at 23°C (42.2 ± 3.0 and 31.5 ± 3.3 g in the H-BMR and L-BMR mice, respectively; F_1,22_ = 5.72, *p* = 0.03; figure 1A), but not at 30°C (39.9 ± 3.2 and 44.9 ± 2.8 g in the H-BMR and L-BMR mice, respectively; F_1,27_ = 1.35, *p* = 0.2). At 30°C, the L-BMR mice raised 42% heavier litters than at 23°C (44.9 ± 2.9 versus 31.5 ± 3.6 g, respectively; F_1,26_ = 8.22, *p* = 0.008), whereas litter mass of the H-BMR mice was not affected by ambient temperature (42.2 ± 2.8 and 39.9 ± 2.9 g at 23 and 30°C, respectively; F_1,23_ = 0.3, *p* = 0.6). Fur shaving had no effect on litter mass within each temperature (F_1,22_ = 0.26, *p* = 0.6 at 23°C and F_1,27_ = 0.08, *p* = 0.8 at 30°C) and within each line (F_1,26_ = 0.005, *p* = 0.9 in the L-BMR line and F_1,23_ = 0.03, *p* = 0.8 in the H-BMR line). The line × fur shaving interaction was also insignificant (*p* > 0.4 in all cases).

**Table 1.** Summary of ANOVA/ANCOVA results of differences in litter mass (g, on day 12 of lactation), maternal *T*_b_ (°C, mean core body temperature over days 12−14 of lactation) and daily energy expenditure (DEE, kJ d^−1^ over days 12−14 of lactation).

main model						
	litter mass^a^		*T*_b_ ^b^		DEE^b^	
	F_d.f._	*p*	F_d.f._	*p*	F_d.f._	*p*
line	0.82 _1,51_	0.4	0.31 _1,18_	0.6	7.53 _1,53_	**0.008 (+)**
ambient temperature	3.39 _1,51_	0.07	5.74 _1,18_	**0.03 (+)**	49.15 _1,53_	**<0.0001 (−)**
fur shaving	0.00 _1,51_	0.9	8.12 _1,18_	**0.01 (−)**	0.66 _1,53_	0.4
line*ambient temperature	6.70 _1,51_	**0.01**	1.55 _1,18_	0.2	0.12 _1,53_	0.7
line*fur shaving	0.04 _1,51_	0.8	0.02 _1,18_	0.9	0.00 _1,53_	0.9

*p*-Values < 0.05 are in bold. Positive signs following *p*-values indicate H-BMR > L BMR and 30°C > 23°C, while negative signs indicate shaved < unshaved and 30°C < 23°C. ANCOVA with litter mass on the 12th day of lactation as a covariate was statistically significant in the case of the ANCOVA of *T*_b_ (*p* = 0.007).

^a^ ANOVA

^b^ ANCOVA

Our results indicate that the litter mass of the H-BMR mice was not affected by the high ambient temperature or fur shaving, suggesting females’ output was not constrained by heat dissipation limits. Litter mass reflects the putative effect of heat dissipation limitation accumulated throughout the lactation period. Thus, our results do not support the HDL predictions, as the litter mass of the H-BMR mice was not reduced at 30°C. Conversely, the exposure of the L-BMR mice to 30°C reduced their thermoregulatory costs and allowed them to raise as heavy litters as those of the H-BMR mice. At 23°C, the L-BMR mice had higher thermoregulatory costs, as Sadowska *et al.* argued [28], which reduced energy available for litter growth. This supposition is supported by the reduction of metabolisable energy intake (MEI) and milk energy output (MEO), which were similar in scope in dams from both lines exposed to 30°C (see the electronic supplementary material, table S1 for details). Since the L-BMR pups grow slower than the H-BMR pups at 23°C [22], a comparable mass of the L-BMR and H-BMR litters at 30°C cannot be attributed to ambient temperature-dependent differences in offspring growth efficiency, but rather to thermoregulatory savings enjoyed by the L-BMR mice.

The above-discussed effect of ambient temperature on litter mass exposed the line-dependent differences in energy expenditure, which are the consequence of both the consistent between-line differences in BMR and SuSMR [20,21] and the resulting 7.6°C difference in lower critical temperatures (LCTs) between lines (31.4°C in the L-BMR versus 23.8°C in the H-BMR mice) [30]. Our results, therefore, demonstrate the suitability of studied lines as an animal model for testing predictions of the HDL hypothesis.

Our results also do not support the HDL prediction that fur shaving increases heat dissipation, as the litter mass of the shaved H-BMR mice lactating at 30°C was not higher than the unshaved dams. Shaving of females’ fur did not affect either litter mass (table 1) or MEI and MEO (the electronic supplementary material, table S1). This result is at odds with that of Simons *et al.* [38], who found that shaved females of common voles (*Microtus arvalis*) raised faster growing offspring than unshaved ones at 30°C. It raises a question of the efficacy of shaving as an effective manipulation of the insulation, which we address below.

### (b) Body temperature

**Figure 2.**
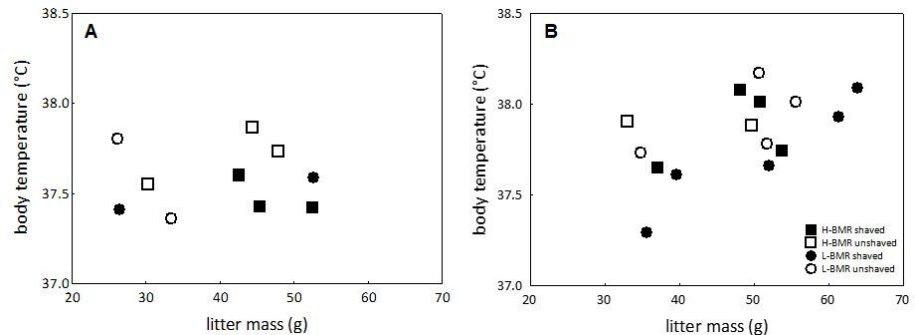
The correlation between litter mass on the 12th day of lactation and mean core maternal *T*_b_ over days 12−14 of lactation in mothers from the L-BMR and H-BMR line raising their pups at temperature of (A) 23°C and (B) 30°C (partial correlation, controlling for the effect of ambient temperature, *r* = 0.31, *p* = 0.41 and *r* = 0.65, *p* = 0.01, respectively).

Our results demonstrate that fur shaving did result in a slight but detectable reduction of body temperature (*T*_b_) in shaved animals (table 1, 37.8 ± 0.06°C versus 37.6 ± 0.05°C in unshaved and shaved dams, respectively). Moreover, at 30°C, we found a positive correlation between litter mass and maternal *T*_b_ (figure 2B, with the highest *T*_b_ observed in the L-BMR mice, raising the heaviest litters). This effect was also discernible as a statistical significance of litter mass in the ANCOVA of *T*_b_ (table 1). However, as discussed above, shaving females did not affect litter mass (table 1). Furthermore, we expected that *T*_b_ would increase in the unshaved and decrease in the shaved H-BMR mice lactating at 30°C. Conversely, since the L-BMR mice lactating at 23°C are burdened with higher thermoregulatory costs, fur shaving should increase these costs, reducing *T*_b_. The above predictions stem from the between-line difference in *T*_b_ being consistently lower in non-lactating mice of the L-BMR line at 23 and 30°C [26]. They were not supported, as the maternal *T*_b_ was affected by fur shaving and ambient temperature but not by line affiliation (table 1, figure 1B).

In our study system, fur shaving affected heat flux in lactating dams (see also, e.g. [39,40]), but not to the extent that it increased litter mass. It is worth noting that the difference in *T*_b_ of dams lactating at 23 and 30°C was roughly equal to 0.2°C (37.6 ± 0.06 and 37.8 ± 0.05°C at 23 and 30°C, respectively) and comparable to the effect of shaving. Our results indicate that even if the HDL constraints are in force, a slight increase in *T*_b_ associated with greater heat loss requirements is well tolerated by lactating females and does not affect litter mass.

### (c) Daily energy expenditure

If the HDL hypothesis holds, constraints on heat dissipation in dams lactating at 30°C should also translate into reduced DEE. Moreover, at 30°C, the shaved mothers should be able to increase their DEE compared with the unshaved ones, and this effect should be more prominent in the H-BMR mice. Our results do not support this prediction. Although exposure to 30°C resulted in the reduction of DEE by approximately 20% in comparison with 23°C (86.8 ± 2.1 and 108.6 ± 2.2 kJ d^−1^, respectively, table 1, figure 1C), it was independent of line affiliation. Irrespective of ambient temperature, the H-BMR mice had higher DEE than the L-BMR mice (102.2 ± 2.2 and 93.2 ± 2.2 kJ d^−1^, respectively, table 1). Fur shaving did not affect DEE (table 1).

Our results are at odds with several studies demonstrating a significant effect of fur shaving on DEE (e.g. [6,16,41]). It is worth noting, however, that those studies showed an increase of DEE of shaved animals exposed to low (2.5 and 4.8°C) or room ambient temperatures (range 20–22°C) rather than an enabling effect of fur shaving on heat dissipation at high ambient temperatures.

It is important to note that the above interpretation of our finding hinges on the assumption that, by the HDL hypothesis, DEE is limited by heat dissipation, and not driven by the pup’s demand for milk [42]. However, our selection for the high BMR indirectly selected for a high pup’s growth rate [22]. It is, therefore, unlikely that, at least in the H-BMR mice, the heat dissipation limit was not reached because of insufficient pup demand for milk production.

## 4. The heat dissipation limitation hypothesis and climate change

Laws of physics dictate that individuals with high energy expenditure may be first compromised by the constraints predicted by the HDL hypothesis [43–45]. Although exposure to 30°C significantly reduced DEE and increased *T*_b_ of the H-BMR females, it did not affect their litter mass—the key proxy of parental effort. This finding is important because BMR is a metabolic trait underlying species’ vulnerability to global warming [45–49]. The H-BMR mouse line surpasses BMR of any other laboratory mice (fig. 3 in [30]). Thus, the energy expenditure of the H-BMR mice and their need for heat dissipation co-generated by this expenditure is equal to or higher than that of other mouse lines. If the litter mass of animals having such a high BMR is not adversely affected by lactation at 30°C, then it is unlikely that the parental effort of small mammal females having lower BMR and lactating at 30°C will be constrained by heat dissipation. However, it is essential to note that female mammals lactating at high temperatures can be constrained not only by heat dissipation, but other factors not directly considered by the HDL hypothesis, chiefly evaporative water loss [50]. To our knowledge, in all laboratory experiments testing the HDL hypothesis, lactating females had unlimited access to water. In contrast, lactation under natural conditions, particularly in arid areas, may be compromised by the dehydration incurred by evaporative cooling before heat dissipation limitations are reached.

Our study demonstrated the still unappreciated significance of the within-species variation of the key metabolic traits, such as BMR, to responses to high ambient temperatures. Paradoxically, individuals characterized by the low BMR (and, therefore, low SuSMR but high LCT) [30] may benefit from the releasing effect of increasing ambient temperature, enabling them to invest more in reproduction and shift natural selection towards reduction of BMR. Therefore, future studies on the HDL hypothesis should focus mainly on this individual variation, which may contribute to the clarification of otherwise equivocal results obtained to date.

## Data Availability

Data are available from the Dryad Digital Repository [51]. Supplementary material is available online [52].
